# A Napkin Point

**Published:** 1904-04

**Authors:** E. G. Hunt


### A NAPKIN POINT.

There is one point that perhaps some of the younger men have not had demonstrated to them—insignificant, perhaps, but useful. If it is desired to keep dry a left lower tooth for ten, fifteen or twenty minutes, act as follows: Fold or roll a napkin into a strip one and a half inches wide and not less than seven or eight inches long. Have the patient raise the tip of the tongue to the roof of the mouth. Place the end of the napkin against the lingual surfaces of the right lower teeth and pass it across the mouth under the tongue to the lingual surfaces of the left lower teeth. Let the tip of the tongue be depressed. Pass the remainder of the napkin back across the mouth from left to right, this time over the tongue. Stand to the right and in front of your patient. Place a cotton roll between the cheek and left upper teeth to occlude the parotid duct. Hold the tongue firmly down against the floor of the mouth with the fingers of the left hand, the thumb being under the chin. The lower fold of napkin occludes the submaxillary and sublingual ducts, and the upper fold enables the operator to hold the tongue down without it slipping. The whole secret of success lies in keeping the tongue firmly down against the floor of the mouth. The patient may at first make unconscious spasmodic efforts to raise the tongue to swallow, but if it is held to place these efforts will cease. For keeping dry the right lower teeth reverse the operation. —*Dr. E. G. Hunt, in Brief.*

